# A review of data needed to parameterize a dynamic model of measles in developing countries

**DOI:** 10.1186/1756-0500-3-75

**Published:** 2010-03-16

**Authors:** Emily K Szusz, Louis P Garrison, Chris T Bauch

**Affiliations:** 1 Department of Mathematics and Statistics, University of Guelph, Guelph, Canada; 2 Department of Pharmacy, University of Washington, Seattle, Washington, USA

## Abstract

**Background:**

Dynamic models of infection transmission can project future disease burden within a population. Few dynamic measles models have been developed for low-income countries, where measles disease burden is highest. Our objective was to review the literature on measles epidemiology in low-income countries, with a particular focus on data that are needed to parameterize dynamic models.

**Methods:**

We included age-stratified case reporting and seroprevalence studies with fair to good sample sizes for mostly urban African and Indian populations. We emphasized studies conducted before widespread immunization. We summarized age-stratified attack rates and seroprevalence profiles across these populations. Using the study data, we fitted a "representative" seroprevalence profile for African and Indian settings. We also used a catalytic model to estimate the age-dependent force of infection for individual African and Indian studies where seroprevalence was surveyed. We used these data to quantify the effects of population density on the basic reproductive number *R*_0_.

**Results:**

The peak attack rate usually occurred at age 1 year in Africa, and 1 to 2 years in India, which is earlier than in developed countries before mass vaccination. Approximately 60% of children were seropositive for measles antibody by age 2 in Africa and India, according to the representative seroprevalence profiles. A statistically significant decline in the force of infection with age was found in 4 of 6 Indian seroprevalence studies, but not in 2 African studies. This implies that the classic threshold result describing the critical proportion immune (*p*_c_) required to eradicate an infectious disease, *p*_c _= 1-1/*R*_0_, may overestimate the required proportion immune to eradicate measles in some developing country populations. A possible, though not statistically significant, positive relation between population density and *R*_0 _for various Indian and African populations was also found. These populations also showed a similar pattern of waning of maternal antibodies. Attack rates in rural Indian populations show little dependence on vaccine coverage or population density compared to urban Indian populations. Estimated *R*_0 _values varied widely across populations which has further implications for measles elimination.

**Conclusions:**

It is possible to develop a broadly informative dynamic model of measles transmission in low-income country settings based on existing literature, though it may be difficult to develop a model that is closely tailored to any given country. Greater efforts to collect data specific to low-income countries would aid in control efforts by allowing highly population-specific models to be developed.

## Background

Measles is a highly contagious disease, spreading especially rapidly in populations that are dense and/or exhibit low immunity [1]. The main natural carrier for measles is humans (though it is possible for some primates to acquire infection) [2], and there are no known long-term reservoirs for the virus [3]. Hence, an effective method of prevention is prophylactic vaccination. Measles can be prevented by a relatively inexpensive and effective vaccine, and yet measles remains one of the leading causes of death in children [4]. Measles caused an estimated 242,000 deaths globally in 2006, 95% of them occurring in countries with high-level poverty and poor health infrastructure [5]. The severity of measles can range from mild symptoms to severe infection, but it is usually the complications following infection that lead to death or disability [3,5]. Though vitamin A supplementation does not seem to have any impact on incidence, duration, or prevalence of infectious diseases [6], it has been shown numerous times to reduce the severity and thus mortality in infectious diseases--including measles and its complications--because of its necessary role in the immune system [2]. Conversely, there have been differing views as to whether or not improving the overall nutritional status of children would reduce the risk of mortality. Review papers by Aaby [7,8] and Singh et al ([9]) found little or no relation between malnutrition and measles related deaths in Africa or India, respectively. However, though Aaby mentions vitamin A as a possible factor in measles related deaths, he says other causes of severe complications may dominate over vitamin A deficiency [7]. Singh et al also recognized the significance of vitamin A but none of the papers included in their review explicitly took vitamin A supplementation into account.

A live attenuated measles vaccine has existed since the mid 1960s [3]. However, strong and coordinated global action to significantly reduce measles mortality did not occur until the launch of the Measles Initiative in 2001. Since then, measles deaths have been reduced by 68% worldwide and 91% in Africa [10]. Much of this reduction has been achieved through Supplementary Immunization Activities (SIAs), which are large-scale campaigns aimed at vaccinating all children under a certain age. In other cases, routine immunization (RI) is being improved either by increasing the coverage of the first dose of measles vaccine, or by introducing a second dose in countries where first-dose coverage is already relatively strong [11]. However, while coverage has reached high levels in many countries, it remains low and relatively stagnant in others. Moreover, the optimal timing of SIAs and the optimal criterion for introducing the second dose of routine vaccination are not known because this depends partly on eventual measles incidence and measles disease burden under the various alternative vaccination strategies being considered. Hence, modelling exercises intended to project optimal vaccination "second opportunity" strategies in this setting may prove valuable.

Although future measles incidence and disease burden cannot be known precisely, they can be projected by use of a dynamic model. Dynamic models (e.g., age-structured compartmental models) incorporate transmission mechanisms and thus can describe how incidence evolves due to changes in factors such as demography and vaccine coverage [12]. Dynamic models allow us to analyse the epidemiology of a disease within a population and experiment with various vaccination strategies "*in silico*" to determine which one would be optimal from a given perspective (e.g. public health, cost-effectiveness).

Ideally, dynamic models are parameterized with data that are specific to the study population. However, measles epidemiology can vary across populations, which in turn provide implications for how vaccination programmes should be designed [13]. In the 1960s, Morley et al recognized and documented important differences between the epidemiology of measles in developing countries compared to measles in the UK and North America, since at least the 1920s [14]. Suggested reasons for this have included differences in living conditions, nutrition, demography (birth rate), and social/cultural behavioural differences [14]. Because of this, models intended for developed countries are not necessarily applicable for developing countries [13]. For example, models developed by Babad et al [15] and Cvjetanovic et al [16] both assume the epidemiology of measles in England and Wales, with a mean age of infection in children of approximately 5 years. This has been thought to reflect the fact that children were relatively isolated from other children until the start of school. In contrast, developing countries have a much lower age at infection, as described by McLean et al [14], and specific social and cultural factors that may explain this have been identified [17], such as larger family sizes and greater mixing from a younger age.

This paper provides a summary of data needed to develop a dynamic model of measles transmission in Indian and African populations, such as the percent infected by age (age-stratified attack rate) and age-stratified seroprevalence profiles. We review included studies individually, discussing their design, limitations, and major conclusions. We use these studies to summarize attack rates and seroprevalence and make deductions about transmissibility in African versus Indian populations. We summarize the broad features of measles epidemiology in low-income countries and compute a "representative" age-stratified seroprevalence profile. We also use the data to quantify the effects of population density on transmissibility measures such as the basic reproductive number *R*_0_. Finally, we use a catalytic model to compute age-dependent force of infection for individual populations where seroprevalence studies were available.

## Methods

Age-stratified attack rates (i.e. the percent of individuals infected in each age group in a given outbreak or over a longer period of time) and seroprevalence are needed to determine susceptibility levels and transmission rates within a population. These measures can vary between rural and urban populations as well as between countries, and over time in a given population. Ideally, one has sufficient data to allow analysis of each population individually; in practice, however, data availability can be limited for any given population [18]. Here, we summarize available data for a number of low-income countries to investigate whether there are any general trends that apply in most or all low-income countries.

The basic reproduction number *R*_0_--the average number of secondary infections produced by an infected person in a totally susceptible population--is an important epidemiological measure that defines a threshold for whether an infectious disease can invade a population. When *R*_0 _is above 1, each infectious person can infect more than one person in a totally susceptible population, and hence it is possible for the infectious disease to invade [19]. Conversely when *R*_0 _is below 1, an infectious disease cannot invade a totally susceptible population. *R*_0 _also plays a role in indicating how easily an endemic infectious disease can be controlled and/or eradicated [19]. *R*_0 _is dependent on population density and this dependence varies between countries [20]. In this paper, we attempt to determine this dependence for Africa and India.

In the following paragraphs, we describe (1) inclusion/exclusion criteria for the literature we used in our analysis, (2) methods for extracting and summarizing age-stratified attack rates for Africa and India, (3) methods for extracting and summarizing age-stratified seroprevalence for Africa and India, and (4) methods for computing the dependence of *R*_0 _on population size.

### Inclusion/Exclusion criteria

To identify relevant literature sources with useful data, Web of Science, Google Scholar, and PubMed were searched using terms including: "case report", "case study", "surveillance", "epidemiology", "seroepidemiology", "antibody", "seroprevalence", and variations on each term combined with the terms: "measles", "Africa" or "India", and sometimes specific country names within Central or Eastern Africa such as "Nigeria", "Niger", and "Uganda". The reference section in each paper was checked and any papers that had referenced them were also researched. We focussed on identifying papers dated before the era of widespread vaccination in Africa and India, i.e., from the 1970s and early 1980s. This is because vaccination influences infection transmission in the population and hence can affect seroprevalence and attack rates [19]. (However, we note that methods do exist for adjusting for such effects). We included several more recent papers as well to analyze the changes in epidemiology as a result of vaccination.

Each source was classified into one of three types: (1) epidemic case reporting, (2) endemic case reporting, and (3) seroprevalence. Epidemic case reporting papers generally describe the percent infected by age during a specific measles outbreak. Conversely, endemic case reporting papers describe reported cases over a longer period of time, during and outside of outbreaks. Case report studies can suffer from under- or over-reporting due to misdiagnosis, unreported cases, or subclinical infection. Seroprevalence studies do not suffer from under/over-reporting subclinical infection, or misdiagnosis and hence can prove very useful when trying to determine what proportion of the population remains susceptible. On the other hand, seroprevalence surveys do not always reflect long-term average conditions in a given population. For instance, a seroprevalence profile obtained just before an epidemic outbreak will differ from one obtained just after an outbreak, and this can influence estimates of the mean age at infection and the force of infection. This is especially a problem for smaller, isolated populations with long inter-epidemic intervals. Moreover, seroprevalence studies cannot distinguish between immunity due to natural infection and immunity due to vaccination. We included seroprevalence publications that used a representative, randomly chosen sample set with a relatively large number of children in each age category being tested. We included case report publications that relied on national surveillance systems or representative community-based surveys.

Many case report studies that we found only used hospital data to estimate incidence or attack rates (eg. [21,22]). Unless some adjustment is made, this may create a sampling bias towards those children who have access to health care or those children sufficiently ill to be admitted to a hospital. Hence, these publications were excluded. Of the seroprevalence studies, many were only concerned with seroconversion rates after vaccination (eg. [23-25]) or collected samples from children and infants attending a health care centre (eg. [26-29]), which again may introduce bias by including only those with access to health care. Hence, these publications were also excluded.

A common occurrence among Indian case report studies was that the authors would report the number of cases in an age group but not give a breakdown by age of the surveyed population, making it impossible to calculate the attack rates (eg. [30-32]). In other publications, children were surveyed regarding the age when they had measles infection, but it was unclear when the population per age group was tabulated, again making attack rates impossible to calculate (eg. [33-35]).

### Reporting and construction of age-stratified attack rate and seroprevalence profiles

We summarized the age-stratified attack rates from most included studies through several figures. The attack rates were either taken directly from the study, when available for each year of age, or found by calculating them from component data reported in the study. If the reported age group spanned more than one year, then the attack rate reported for the whole age group was also assumed to represent the attack rate for each individual year of age in that age group.

Similarly, from the seroprevalence studies, the percent of children seropositive for measles antibody was taken directly from the data, when available for each year of age. If the reported percent seropositive in an age group spanned more than one year, then the seropositive value was assumed to represent the percent seropositive of the midpoint age of the age group only.

To fit a "representative" seroprevalence profile, we first constructed an average profile from the surveys reviewed as follows. If only one of the reviewed studies reported seroprevalence at a given age, then that was used as the seroprevalence of the average profile at that age. If more than one reviewed study reported seroprevalence at a given age, then the average seroprevalence of those studies--weighted by their sample size--was used as the seroprevalence of the average profile at that age according to the equation:(1)

where *S*_i _is the average seroprevalence in age class *i*, *s*_ij _is the seroprevalence in age class *i *from study *j*, *n*_ij _is the number of samples in age class *i *of study *j*, and *N*_i _= Σ_*j*_*n*_*ij*_. Finally, if no reviewed study reported seroprevalence at a given age, then the seroprevalence of the average profile was not given for that age. Below age 1, averaged seroprevalence data points were sought for 3, 6, and 9 months (or as close to that as possible), and it was assumed that all newborns had maternal immunity. Above and including age 1, averaged seroprevalence data points were sought for each year, although they were not always available, especially for some older ages where no included studies reported samples from that age.

This average seroprevalence profile was then fitted to the following equation to generate a "representative" or "typical" age-stratified seroprevalence profile:(2)

where *S*(a) is the percent seropositive in age *a*, and *k*_1_, *k*_2 _and *k*_3 _are constants. The last term *exp*(-*k*_3_*a*) in Equation (2) describes an exponentially declining curve that captures the decline in seroprevalence attributable to maternal immunity in the first year of life. The rest of Equation (2) describes a curve that captures seroprevalence attributable to infection, where *k*_1 _and *k*_2 _control how quickly seroprevalence increases with age and how high seroprevalence can become in the oldest age groups. For realistic values of *k*_1 _and *k*_2_, one obtains an S-shaped seroprevalence profile with an inflection point at young ages, that approaches 100% seroprevalence asymptotically. By dropping the maternal immunity term in Equation (2) one can also define the seroprevalence attributable to infection as:(3)

These equations were adapted from the approach described in Ref [36] and references therein. The values of *k*_1_, *k*_2 _and *k*_3 _that yielded the smallest ordinary least squares error between *S*(a) and the average seroprevalence profile were deemed optimal and used to construct the representative seroprevalence profile. The squared error at each age was weighted according to how many adjacent ages (+/- 1 year) had known average seroprevalence data points, to mitigate over-sampling of age ranges for which there were many available average seroprevalence values. Because Equation (2) accounts for decay of maternal antibodies, we can fit it to seroprevalence data for all age categories, including those below 1 year of age. Although Equation (1) adjusts for variable sample sizes across studies within a given age class, we do not adjust for variable sample sizes across age classes when fitting Equation (2). We note that this introduces the potential for wayward estimates of seroprevalence to influence the best-fit representative seroprevalence profile.

Plots of age-stratified attack rates from included studies were constructed for both India and Africa. Separate plots were constructed for epidemic versus endemic reports. Plots were also constructed from included studies for age-stratified seroprevalence surveys for India and Africa. The representative seroprevalence profile from Equation (2) was included on the same plot.

### Estimation of force of infection

To estimate the force of infection from the average seroprevalence profiles for Africa and India, we formulated several functions for an age-dependent force of infection, modelled the seroprevalence profiles resulting from those functional forms, and fitted the resulting seroprevalence model to the average seroprevalence profiles for India and Africa. A catalytic modelling approach adjusting for the presence of maternal antibodies allows us to relate the force of infection *λ *(*a*) at age *a *to seroprevalence via:(4)

where *k*_3 _is the maternal immunity component emerging from Equation (2). Equation (4) can be discretized with respect to age, giving:(5)

where Δa is a (very small) age increment from *j*-1 to *j*, *S*_j _is seroprevalence at age *j*, and *λ*_j _is the force of infection at age *j*. Equation (5) can be solved for *S*_j _yielding(6)

From Equation (6), the seroprevalence arising from any assumed force of infection can be derived. The four functional forms for the force of infection that were tested were:(7)

where *S*(*a*) and *S*_inf_(*a*) in Equation (10) are taken from Equations (2) and (3) according to the seroprevalence model of Ref [36].

Values of *k*_1_, *k*_2 _(where applicable), and *k*_3 _were tested with fine resolution across broad ranges and the sum-of-squares error between the seroprevalence computed from Equation (6) and the average seroprevalence profiles for India and Africa was computed. The criterion for best fitting parameters was the minimization of sum-of-squares error. The model parsimony of the linear and exponential forms relative to the constant forms were analyzed via an F-test in order to test the hypothesis that the force of infection declines with age. A declining force of infection with age would indicate that the eradication equation *p*_c _= 1-1/*R*_0 _overestimates the required proportion immune for eradication in those populations [19]. The F-ratio is the ratio of the increase in the sum-of-squares error (of the simpler model relative to the more complex model) to the increase in the degrees of freedom (of the more complex model relative to the simpler model). An F-ratio greater than 1 indicates that the more complex model may be parsimonious.

This method of computing the force of infection was repeated for each individual age-stratified seroprevalence profile from the included Indian and African studies, with the exceptions of Refs [37] and [38] in African populations, due to lack of data points below 1 year of age in those surveys. Hence, we computed force of infection for 6 studies in Indian populations and 2 studies in African populations, for the exponential and constant functional forms.

### Estimation of *R*_0 _and relation to population size

Studies have shown that *R*_0 _increases with increasing population density, particularly via:(6a)

where *N *is the total population, *K *is a scaling constant, and 0 ≤ *c *≤ 1 is a constant that determines the impact of population density on *R*_0 _[20].

The relation between *R*_0 _and population size was determined in both African and Indian populations using case reporting and seroprevalence data. In Africa, we estimated *R*_0 _values for large cities or rural areas in 8 different countries, whereas in India, *R*_0 _values were estimated for 11 different urban or rural regions. In one African study, the *R*_0 _value was estimated by averaging over a statistical description of *R*_0 _values reported directly by the authors and so no further calculation was necessary. For all others, the *R*_0 _value was estimated using the formula:(7a)

where *G *is the inverse of the per capita birth rate, *A *is the age at infection in years assuming no vaccination within the population, and *D *is the average duration of maternal antibodies [14]. *D *= 3.3 months was estimated from a study by Hartter et al [39]. *G *was estimated from demographic data made available in the study or, if not available in the study, from other various sources.

The mean age at infection *A *was calculated in two different ways depending on what data were available in the study. Both methods assume that the populations have experienced no previous history of vaccination, which of course is only approximately true in most cases. Therefore, the following methods estimate the mean age at infection in a partially vaccinated population, *A'*. If the force of infection (*λ*) was known through a pre-existing estimate, *A' *was estimated via [19]:(8a)

If *λ *was not given, then *A' *was estimated via:(9a)

where *r*_*i *_is the attack rate at age *a*_*i*_. We note that it is possible to underestimate *A' *using this formula if attack rates at higher ages are not given [14,18]. Each age was taken at the midpoint of the year (i.e. *a*_*i *_= 0.5 yr, 1.5 yrs, 2.5 yrs, etc). For case report studies, attack rates were determined as before. For seroprevalence studies, the attack rate at age *a*_*i *_above one year of age was estimated as:(10a)

where *S*_*inf*, *i *_is the proportion seropositive at age *a*_*i*_. The values of *S*_inf, i _were the best-fit values of Equation (3) to the data from each individual study, above the age of 1.

Since vaccination can increase the mean age at infection, the values of *A' *computed from a population where vaccination is occurring must be adjusted before Equation (7) can be applied. To adjust for vaccination, we used the following relation between the mean age at infection *A' *with vaccination coverage and the mean age at infection *A *without vaccination:(11a)

where *p *is the percent of the study population with vaccine derived immunity [19]. To find *p*, we first sought vaccination coverage data reported specifically for the populations studied. If this was not given, *p *was found using the total vaccination coverage by country [40] for the study year and the number of years previous equal to the number of age groups sampled (if possible) and averaged. We assumed a vaccine efficacy of 85% [41]. Thus *p *is just the product of the average vaccine coverage and vaccine efficacy. The resulting values of *A *were used in Equation (7).

Thus, with estimated values of *R*_0 _for each population sampled, and with the corresponding population size *N*, we fitted a linear regression to Equation (6), rescaled on a ln-ln plot, and used ordinary least squares criterion to obtain the best-fit values for *c *and *K*.

## Results

Tables summarizing the studies that were included in the analysis are given in Additional Files 1, 2, 3 and 4. The first two files describe all included case reporting studies for Africa and India respectively, and the second two files describe all included seroprevalence studies for Africa and India respectively. For each country, the tables describe the population studied, the year of study, type of study, study objectives, major conclusions, and limitations. In the following paragraphs we describe the studies. The review of these studies is broken down by region (Indian versus African populations) and study type (case reporting versus seroprevalence). In the latter part of the Results we present the analysis of the dependence of *R*_0 _on population size.

### Case report analysis: Africa

**Figure 1:**
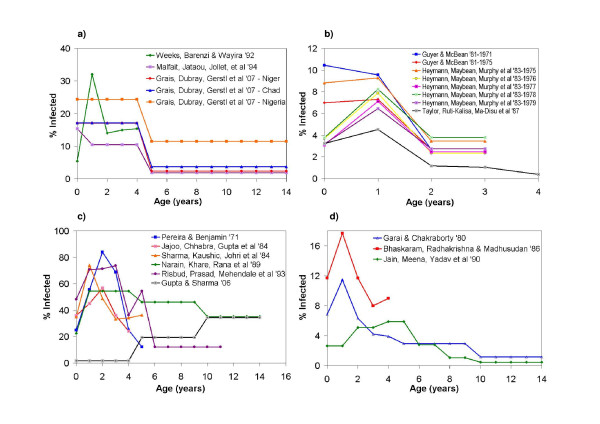
**Percent infected by age for outbreak and endemic studies**. The value shown at age 0 actually represents all children under the age of 1 year. Flat lines indicate an attack rate for an age group that spans more than one age. a) Epidemic studies in Africa. b) Endemic studies in Africa. c) Epidemic studies in India. d) Endemic studies in India.

The age-stratified attack rates for African populations are summarized in Figure 1a (epidemic case reports) and 1b (endemic case reports). The age of peak attack rates is 1 year in almost all studies where sufficiently fine age stratification allows this observation to be made. As already noted, this is a much younger age than observed in the "developed" world before the advent of mass vaccination in the middle 20^th ^century [14,18].

In Africa, three epidemic studies were analyzed and all took place within an urban setting. An epidemic took place in Kampala, Uganda [42] and Niamey, Niger [43] within the same year but the epidemics exhibited different characteristics. Though Kampala had almost twice the population of Niamey and lower vaccine coverage, the highest attack rate was found in the 12-23 month age group, compared to 6-8 months of age in Niamey. However, Niamey had an overall lower total percent infected in children under 5 years than in Kampala (11.65% vs. 16.94%). In 2004, outbreaks were investigated in representative regions in three separate countries: Nigeria, Niger, and Chad [44]. Comparing attack rates in Niger between this epidemic and the early epidemic in 1990 [43], we see that the reported attack rate under age 5 is higher. However, out of the three countries investigated by Grais et al [44], Niger had the lowest overall attach rate in children less than 5 years.

The variation in epidemic patterns between and within countries can be due to a variety of factors including geographical location, crowding effects, availability of health care, and changing birth rates which would change the rate of recruitment of susceptible individuals [45]. And even though vaccine coverage may seem quite high (73% in Niamey, 1990), there may be a high vaccine failure rate due to poor maintenance of the cold chain or because vaccinations were given to children already immune, or were given at the incorrect age [46].

Four endemic case reporting publications were also reviewed, three within an urban setting [46-48] and one in a rural setting [49]. In both the rural and urban settings, the highest attack rate was seen within the 6-11 month age group except in one study which reported the highest attack rate in children 9-23 months [47]. The papers by Guyer et al [46] and Heymann et al [47] combined describe the epidemiology of measles over a period of ten years within the city of Yaoundé, Cameroon. Yaoundé experienced a continuous decline in attack rates in all age groups from 1972 to 1979, even though surveillance was increased in 1974 and then again in 1977. There was a large decrease in attack rates in the younger age groups, but also a smaller decrease in older age groups, suggesting a possible herd immunity effect. Heymann et al report constant vaccination coverage of roughly 40% over most of this time; consistent vaccine coverage may have led to the decline in attack rate. The discrepancy between the two papers for attack rates in 1975 could be due to how the authors estimated the population per age group. Taylor et al [48] reported on Kinshasa, Zaire (now known as the Democratic Republic of the Congo) several years after Heymann et al published their paper. The cumulative percent infected calculated from their data showed a slightly higher percentage in children less than 1 year but lower in all age groups above that. Though Kinshasa had a much higher population than Yaoundé, they had a continual increase in vaccination coverage from 1977 to 1983 which probably allowed for a decrease in attack rate to those comparable to Yaoundé. However, Taylor et al explain that there was non-uniform vaccination throughout the city, which left pockets of susceptible individuals, allowing for continuing transmission despite overall high vaccine coverage. Furthermore, children under 9 months may have served as a reservoir for measles transmission because most were too young to be efficaciously vaccinated but had a high incidence rate. Although vaccinating at a younger age may suggest itself as a strategy to combat this, the vaccine is significantly less efficacious below 9 months of age due to interference from maternal antibodies, and vaccinating to sufficient levels in older age groups can provide herd immunity that protects younger age groups [43].

### Case report analysis: India

Figure 1c (epidemic) and 1d (endemic) summarize the age-stratified attack rates for various Indian populations. From the studies that are stratified by year, the highest attack rate lies roughly between 1 and 2 years of age, which is slightly higher than that noted in the African populations but still lower than in developed countries in the mid-twentieth century. Furthermore, the most recent studies in each figure show that the peak attack rates have shifted to the older age groups. This may reflect changing demographics, social circumstances, or improving vaccine coverage.

Three endemic papers were analyzed for India. Garai et al [50] and Jain et al [51] both conducted community-based surveys in rural areas over a period of three years. Of the two, Jain et al indicated a distinctive lower cumulative % infected at every age up to age 14 yrs. Many factors could have influenced this, such as geographic location or timing of epidemics, but because both studies spanned three years a more likely cause is a higher vaccination coverage seen in the population studied by Jain et al. Though vaccination coverage was not mentioned in either paper, we know that mass routine immunization did not start until the mid 1980s so it is quite possible that Garai et al studied an almost vaccine free population while Jain et al were able to study a population with at least some vaccination. Bhaskaram et al [52] showed a higher cumulative % infected despite being conducted in between the previous two studies. However, their study was conducted in an urban slum and so crowding effects may have caused an increase in the number of children infected. Also, the study was conducted over a period of one year, so it is possible that it was an epidemic year.

We were able to find six outbreak papers for India [53-58], all within a rural setting with minimal or no vaccination at all, except Gupta et al [58]. There does not seem to be variation in the attack rates across varying vaccination coverage levels or population sizes. This is also seen in the paper by Narain et al [56] who surveyed 13 unvaccinated villages, where both large and small villages experienced high attack rates. Isolated populations can experience only intermittent but large outbreaks of measles that may be more difficult to predict [59], so the apparent lack of clear dependence on vaccine coverage or population sizes may reflect lesser predictability of measles outbreaks in rural populations. In all papers except Gupta et al, the highest attack rate during the outbreak was in children below the age of 4 years. Gupta et al was the only paper to have an increasing percent infected by age group, the lowest being in children 0-4 years and the highest being in children 10-14 years.

Though Chand et al [60] was not included in the table, we felt it necessary to discuss as it does give overall attack rates for children under 14 years of age and for the entire population of a representative village over a period of thirteen years. Before 1980, epidemics occurred every 2 years at varying levels of intensity, but after 1980, the inter-epidemic period lengthened to 3 years. This transition from outbreaks every 2 years to outbreaks every 3 years may reflect the introduction of vaccination [61]. It can also be seen that during the last 3 years of the study, the fluctuations in incidence rates becomes less dramatic. The authors suggest that these changes in the outbreak patterns of the disease were a result of the Measles Vaccination Programme.

### Seroprevalence analysis: Africa

**Figure 2:**
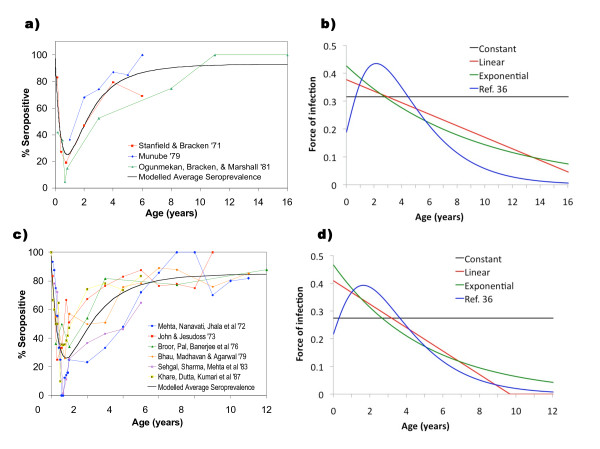
**Seroprevalence and force of infection by age**. a) Representative seroprevalence profile for Africa. The parameters for the best-fit average seroprevalence profile are *k*_1 _= 0.56, *k*_2 _= 0.46 and *k*_3 _= 2.64. b) Force of infection in Africa, computed from representative seroprevalence profile for Africa (panel 2a) using Equation (5). c) Representative seroprevalence profile for India. The parameters for the best-fit average seroprevalence profile are *k*_1 _= 0.63, *k*_2 _= 0.58, *k*_3 _= 2.62. d) Force of infection in India, computed from representative seroprevalence profile for India (panel 2c) using Equation (5).

We identified five studies that give a reasonable to excellent age breakdown of seroprevalence in African populations; however we only included the three oldest in Figure 2a since they were likely to have limited vaccination coverage and thus reflect exposure to natural infection rather than immunization [62-64]. These three were thus used to estimate an average seroprevalence profile. The average seroprevalence profile computed from these studies indicates that about 60% of children exhibited seropositivity for measles antibody by the age of 2. Figure 2b shows the age-dependent force of infection as computed from the representative seroprevalence profile in Figure 2a, using Equation (5).

There was considerable variation in seroprevalence by age across the studies although it was quite evident that the two more recent studies [60,37] showed a much higher seroprevalence rate among children over 1 year old. This is most likely due to increased vaccination (~80% in Addis Ababa and national coverage of 55% in Eritrea prior to when the study was carried out), but perhaps also the fact that both were conducted in an urban setting where crowding effects occur. The two older urban studies [63,64] both show a low seropositivity in children aged 1 to 5 years, but Munube et al [38] show a high seropositivity by age 5 comparable to that reported by Enqusellassie et al [37]. This could be due to sampling location or the fact that their sample size was smaller than all the others. Unfortunately, vaccine coverage was not available in most studies, so we could not compare vaccine coverage to seroprevalence more systematically. All of these studies were in urban populations, except for a district-wide survey in Busoga district, Uganda, which included rural populations [38]. As noted in the Methods section, seroprevalence profiles can be less representative of average conditions in cases where measles epidemiology is characterized by intermittent outbreaks, as might occur for some isolated rural populations.

### Seroprevalence analysis: India

We identified 6 seroprevalence studies in Indian populations [65-70], all conducted in an urban setting and published in the 1970s or early 1980s (Figure 2c). Seroprevalence increases less quickly with age than in the African studies, as seen in the modelled average seroprevalence curves. This suggests that the force of infection is higher at younger ages in African populations. However, the lowest seroprevalence occurs at the age of 8 months in both India and Africa, demonstrating similar waning patterns of maternal antibodies. Because all these studies were in urban populations, the lack of representativeness of the seroprevalence profile that can be caused by intermittent outbreaks in small, isolated populations should not be an issue here (see Methods). Figure 2d shows the age-dependent force of infection as computed from the representative seroprevalence profile in Figure 2c, using Equation (5).

Seroprevalence data do not usually distinguish between immunity from infection and immunity from vaccination but since mass routine vaccination did not start until the mid 1980s [40], the papers we have give us a good picture of how prevalent measles would be without vaccination, at least within an urban setting during those decades.

In almost all of the studies in Indian populations, roughly 80% of children are seropositive by age 6 years. The exception is Sehgal et al. [69], which incidentally had the highest sample size. In most papers, seroprevalence could either increase or decrease from one age to the next. This could be evidence of a cohort effect [71,72], but is more likely a sample size effect. Interestingly, Sehgal et al was the only paper not to exhibit this effect, again perhaps because of its large sample size. Khare et al [70] report a lower seroprevalence in children under 9 months but a higher seroprevalence above 9 months, compared to other studies. This study is relatively recent and thus its higher seroprevalence at younger ages could reflect the initial effects of vaccination. Vaccination also shifts incidence to older children due to herd immunity, which protects younger children from acquiring measles infection. From the seroprevalence profile, we see that below the age of 8 months, the majority of the seroprevalence will be from maternal antibodies that continually decline. The rise again in seroprevalence after 8 months is then due to the acquisition of measles infection, or vaccination.

### Estimation of force of infection

Table 1 gives the best-fitting parameter values for the four force of infection functions fitted to the average seroprevalence profiles for Africa and India. Also shown are the results of the F-test for comparing the exponential and linear force of infection functions to the constant force of infection function. The minimum sum-of-squares error is smaller under the exponential and linear functions (wherein the force of infection declines with age at best-fit parameters for both functions) than the constant function. Also, the F-ratios are larger than 1 for the linear and exponential functions for the Indian seroprevalence profile, implying that the linear and exponential functions with declining force of infection with age are more parsimonious (explain the data better) than the constant function where force of infection does not decline with age. These results imply that the force of infection may decline with age in Indian populations of this time period. F-ratios for the African seroprevalence profile are smaller than 1, hence there is not strong evidence for a declining force of infection in the surveyed African populations of that time. The best-fitting force of infection functions for both African and Indian seroprevalence profiles appear in Figure 2b, d.

**Table 1:** Fit of seroprevalence data to seroprevalence model under different force of infection (FOI) functions

Africa					
**FOI** **Function**	**Best-fit *k*_1_**	**Best-fit *k*_2_**	**Best-fit *k*_3_**	**Least sum-of-squares**	**F ratio**

Constant	0.315	--*	3.59	0.042	--
Linear	0.377	0.055	3.56	0.036	0.33
Exponential	0.427	0.108	3.58	0.034	0.48
Unimodal	0.532	0.452	2.88	0.044	-- **

**India**					

**FOI** **Function**	**Best-fit *k*_1_**	**Best-fit *k*_2_**	**Best-fit *k*_3_**	**Least sum-of-squares**	**F ratio**

Constant	0.275	--*	2.97	0.0185	--
Linear	0.410	0.103	3.16	0.0058	4.33
Exponential	0.468	0.199	3.26	0.0059	4.25
Unimodal	0.598	0.568	2.81	0.0077	2.76

*No k_2 _parameter in function.

** An F ratio was not computed because least sum-of-squares was greater than the constant case.

Table 2 gives the results of the F-test for the 8 individual seroprevalence surveys, for comparing the exponential function to the constant function only. The F-ratio is greater than 1 in 5 of the 6 Indian seroprevalence surveys, but the F-ratio is less than 1 in both of the 2 African seroprevalence surveys. This again suggests that the force of infection declines with age in these Indian populations at the time of the survey.

**Table 2:** F test results from individual seroprevalence studies for exponential versus constant force of infection function

Study	City	F ratio
Mehta et al 1972 [65]	Bombay, India	0
John and Jesudoss 1973 [66]	Vellore, India	2.44
Broor et al 1976 [67]	Chandigarh, India	3.86
Bhau et al 1979 [68]	Pondicherry, India	2.62
Sehgal et al 1983 [69]	Delhi/Alwar, India	0
Khare et al 1987 [70]	Delhi, India	1.25
Stanfield and Bracken 1971 [62]	Kampala, Uganda	0.46
Ogunmekan et al 1981 [63]	Lagos, Nigeria	0

The unimodal force of infection predicts an increase in the force of infection with age, followed by a decrease. We note that the eradication equation *p*_c _= 1-1/*R*_0 _overestimates the critical proportion of immune only if the force of infection declines beyond the mean age at infection. For the exponential and linear functions which decline monotonically with age, this condition is automatically satisfied. However, for the unimodal function, it is only satisfied if the mean age at infection exceeds the age of peak force of infection. At the best-fit parameter values for the unimodal function for the 6 Indian and 2 African seroprevalence surveys of Table 2, this condition is always satisfied (results not shown).

### *R*_0 _estimates: Africa

The following populations in Africa were used to determine the relationship between *R*_0 _and population size: Niakher (Senegal, 1983-2001) [73], Niamey (Niger, 2003-2004) [74], Machakos (Kenya, 1984) [17], Moshi (Tanzania, 1984) [17], Kinshasa (Zaire, 1983) [48], Kampala (Uganda, 1990) [42], Yaoundé (Cameroon, 1971, 1975) [46], and Lusaka (Zambia, 1996-1999) [75].

**Figure 3:**
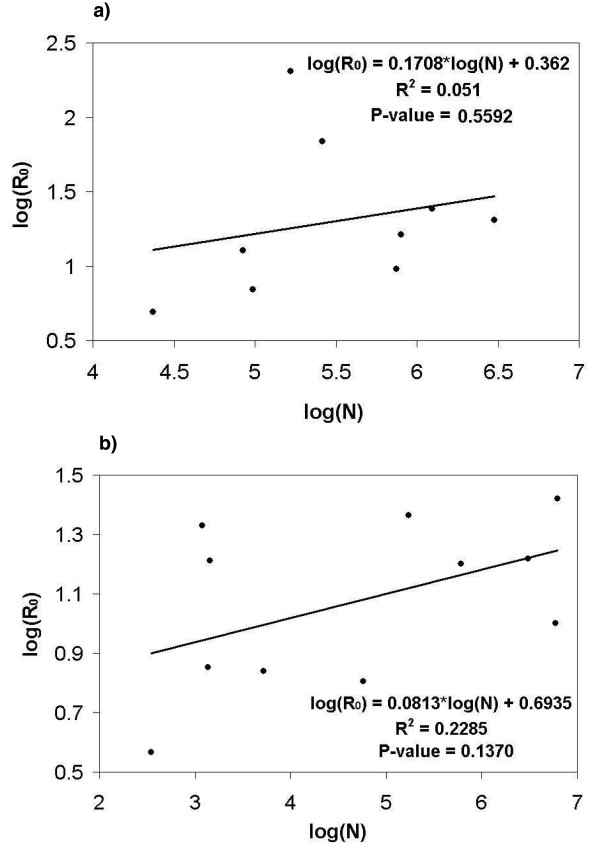
**Plot of log(*R*_0_) versus log(*N*) to determine the value of *c***. a) Linear regression for Africa. R^2 ^= 0.051, P < 0.6. b) Linear regression for India. R^2 ^= 0.2285, P < 0.14.

Data required to calculate *R*_0 _are summarized in Table 3 and a log-log plot of *R*_0 _versus *N *is given in Figure 3a whose slope is the constant *c *in Equation (6). The first two studies were the only two to provide *R*_0 _estimates in the publication or estimates of the mean age at infection. The *R*_0 _= 9.6 value for Niamey was found by averaging over all *R*_0 _values reported for various districts of the city. For Niakher, Broutin et al reported a mean age at infection (with no vaccination coverage) of 4.6 years and a per capita birth rate of 47/1000 giving an *R*_0 _value of 4.9. (The authors also estimated *R*_0 _but did not adjust for maternal immunity).

**Table 3:** Summary of key values needed to determine *R*_0 _values for Africa

City/Country	Birth Rate	*p*	*A'*	*A*	*N*	*R*_0_
Niamey/Niger [74]	n/a*	n/a	n/a	n/a	750000	9.5641
Niakher/Senegal [73]	n/a*	n/a	n/a	n/a	23413	4.9194
Machakos/Kenya [17]	43/1000 [77]	0.2784	2.9070	2.0977	84320 [78]	12.7587
Moshi/Tanzania [17]	50.5/1000 [79]	0.0000	3.1153	3.1153	96838 [78]	6.9719
Kinshasa/Zaire [46]	49/1000 [80]	0.2040	1.6078	1.2798	3000000	20.3101
Kampala/Uganda [40]	50.1/1000 [79]	0.4080	2.5355	1.5010	800000	16.2804
Yaounde/Cameroon 1971 [44]	45/1000	0.6053	0.9736	0.3843	166000	203.3308
Yaounde/Cameroon 1975 [44]	45/1000	0.4316	1.0522	0.5981	260000	68.7812
Lusaka/Zambia [75]	40/1000	0.7395	5.0000	1.3025	1240000	24.3309

*n/a = not applicable since authors report *A *or *R*_0 _directly

Equation (8) was used to find *A' *in Machakos, Moshi and Lusaka, and Equation (9) was used for the rest of the studies. Seroprevalence studies were not included due to the relatively few studies with sufficiently good age stratification to allow computation of *A*. The study by Enquselassie et al [37] was also not included because their data incorporated a high proportion of vaccinated individuals in all age groups. The recommended age for vaccination is 9 months and so vaccinated populations have a very high seropositivity rate from a very young age which would misrepresent the actual seroprevalence due to natural infection. Birth rates were reported in the studies for Yaoundé and Lusaka, but for the most part, birth rates were found from various other sources. Vaccination coverage for each study population was reported in all papers except Taylor et al and Scott et al. For those two studies, estimates from the World Health Organization (WHO) website were used to calculate *p*. *R*_0 _values were calculated and reported in Table 3. The unusually high value of *R*_0 _for Yaoundé, Cameroon, 1971, may reflect the fact that only cases below the age of 3 were reported (though these constituted 90% of all cases, according to the authors). The best fit value of a linear regression to a plot of log(*R*_0_) versus log(*N*) yielded *c *= 0.1708, the *R*^2 ^value was 0.051, and the P < 0.6. This P-value suggests that it is more likely that there is no relation between population and *R*_0_; however, we sampled over many different African countries so it is possible that this relation would be stronger within individual countries.

### *R*_0 _estimates: India

We estimated *R*_0 _values for 11 different regions in India: Sathuvachari village (Tamil Nadu, 1969) [53], Ramgarh village (Rajasthan, 1986-1988) [51], Hyderbad city (Andhra Pradesh, 1982-1983) [52], Hooghly District (West Bengal, 1976-1978) [50], 13 villages of Tehri Garhwal (Uttar Pradesh, 1986) [56], Dumehar village (Himachal Pradesh, 2004) [58], Barbadi (Maharashtra, 1982) [54], Bombay (Maharashtra, 1972) [65], Vellore (Tamil Nadu, 1973) [66], Pondicherry (Puducherry, 1979) [68], and Delhi (1984-1985) [70].

The key values needed to determine *R*_0 _values are summarized in Table 4 and Figure 3b gives the log-log plot of *R*_0 _versus *N*. For every study population, Equation (9) was used to determine the mean age at infection with vaccination. Equation (11) was then applied to find *A*. The first 7 studies were case report studies and so the mean age at infection was calculated directly from the attack rates. The last 4 were seroprevalence studies so Equation (3) was used first to fit the data to ensure non-negative attack rates. Birth rates were obtained through correspondence with a member of the Population Reference Bureau. The birth rates were given by state and so it was assumed these birth rates accurately represented the birth rates for each village or city studied. The duration of immunity due to maternal antibodies was assumed to be the same as that in Africa. This was justified earlier in the analysis of seroprevalence in India where it was noted that both India and Africa have similar waning patterns of maternal antibodies. Only 3 out of the 11 studies reported vaccination coverage. For all others it was either assumed that there was no vaccination coverage due to the timing of the study or it was specifically stated in the paper. Populations were either given in the study or found from websites as close to the time of the study as possible. The resulting best-fit value of *c *was 0.0813, the *R*^2 ^value was 0.2285, and the P < 0.14. This P-value is lower than that for Africa, suggesting that it is likely that the relationship between *R*_0 _and population is stronger within a country [20], despite the heterogeneity in conditions across Indian states.

**Table 4:** Summary of key values needed to determine *R*_0 _values for India

City/Region	Birth Rate	*p*	*A'*	*A*	*N*	*R*_0_
Sathuvachari [51]	31.4*	0.2010	2.2401	1.7898	1200	21.4339
Ramgarh [49]	36.2	0.0468	4.4506	4.2426	5258	6.9243
Hyderbad [50]	30.8	0	2.2401	2.2401	3,043,896 [78]	16.5221
Hooghly District [48]	35.2**	0	4.7190	4.7190	57267	6.3927
Tehri Garhwal [54]	37.5	0	7.4924	7.4924	349	3.6948
Dumehar village [56]	19.2	0.2870	10.6615	7.6017	1360	7.1087
Barbadi [52]	29.8	0	2.3353	2.3353	1434	16.2875
Bombay [65]	32.2	0	3.3733	3.3733	5,970,575 [81]	10.0237
Vellore [66]	30.0	0	1.7155	1.7155	171157 [78]	23.1407
Pondicherry [68]	29.0	0	2.4465	2.4465	604,471 [78]	15.8800
Delhi [70]	31.0	0	1.5001	1.5001	6,220,406 [78]	26.3316

*birth rate was listed for 1971, study was done in 1969

**value was extrapolated from graph of birth rate vs. year

***values for *A' *differ slightly from those in Table 3 due to differing age ranges used in calculation

## Discussion

Although the quality of data for low-income countries varies widely, as do epidemiological patterns, several broad trends emerge from this literature review and analysis. Firstly, this review confirms previous findings that the mean age of infection and age of peak attack rate, are significantly lower in African and Indian populations than was common in Western countries before the advent of mass vaccination [14,18]. This kind of information can be used to parameterize dynamic models for low-income countries, and suggests that some interesting differences may emerge between model projections for Western countries and those for low income countries.

A classical result of epidemiological theory is that the critical proportion immune required to eradicate an infectious disease equals 1 - 1/*R*_0_. For populations where the force of infection declines with age, this is an overestimate of the critical threshold required to eradicate a disease, because it assumes homogeneous mixing across ages [19]. Intuitively, this effect occurs because vaccination increases the mean age at infection, so if the force of infection decreases with age, the impact of vaccination is multiplied as vaccination pushes infection events to older ages where the force of infection is lower [19]. The results of Tables 1 and 2 suggest that the eradication equation *p*_c _= 1-1/*R*_0 _may overestimate the proportion of individuals that need to be vaccinated in order to eradicate measles in at least some populations low-income countries. This issue may not be important for models of measles in developed Western populations, where the force of infection climbs, peaks between ages 5 and 15, and then declines, making it difficult to know *a priori *the direction of bias introduced by using the equation *p*_c _= 1 - 1/*R*_0_. However, we also note that other aspects of real populations, such as spatial and social heterogeneity, could conceivably make measles eradication more difficult than suggested by either age-structured or homogeneous compartmental models.

As vaccination decreases transmission and thereby increases the mean age at infection, transmission dynamics in older age classes can become an important factor in determining the effectiveness of immunization programmes. Because of this, reliable data on seroprevalence in older age classes is needed to accurately estimate force of infection in these classes. However, obtaining such reliable data can be difficult because the vast majority of individuals are infected at a young age, which introduces sample size issues for inferring force of infection at older ages. In these cases, quality of the data becomes more important. However some assays may have poor sensitivity which could lead to underestimating seroprevalence. If the force of infection is estimated using insufficient or poor quality data in older age classes, and is used to parameterize the age-specific transmission rates of an age-structured compartmental model [19], there is a significant possibility of erroneous predictions.

This literature review produced *R*_0 _estimates that vary widely across various populations, from 4 to 26. Although the data upon which these estimates are based are generally limited in quality, estimates from other populations for which better data are available also indicate significant variability in *R*_0 _estimates for measles [19], and so this outcome is not unexpected.

The attack rates in African populations studied peak at age 1 for both rural and urban areas, although studies from later years when vaccination coverage is higher tend to indicate later peaks. According to the average seroprevalence profile estimated with a model from populations with little or no vaccination, roughly 60% of children are seropositive by age 2.

In the Indian populations studied, the attack rates peak between 1 and 2 years, which is somewhat higher than for African populations studied. Newer studies also indicate a shift in high attack rates to older children. The average seroprevalence profile calculated for India also shows that roughly 55% of children are seropositive by age 2, which is slightly lower than for the average African seroprevalence. Hence, both case reporting and seroprevalence surveys suggest a lower force of infection in most Indian populations than in most African populations of the time.

A distinct difference in epidemiological patterns between rural and urban areas also emerged in this review. The studies in rural areas are more highly variable in terms of attack rates, often with no obvious dependence on population size or vaccination coverage. This is consistent with the projections of dynamic measles transmission models that exhibit highly variable, apparently unpredictable outbreaks in small isolated populations, but relatively more regular patterns in dense urban populations [59].

Though case report studies are relatively easy to carry out and can give a general picture of the epidemiology of measles, they also exhibit several general weaknesses. For instance, Munube et al [38] point out that many people believe measles can be acquired more than once in a lifetime. This belief stems mainly from the fact that young children are more prone to diseases with similar symptoms to measles, resulting in misdiagnosis in studies relying upon self report. (This can also mean that children are likely to go unvaccinated because it appears that natural infection cannot cause permanent immunity). This concern has also been discussed in a paper by DeFrancisco et al who conducted a study in rural Bangladesh [76]. They explain that over-reporting is quite common because parents or guardians misdiagnose their child's disease. Conversely, the Indian studies we reviewed usually exhibited better reliability of case reports: Mehta et al [65] and Khare et al [70] both show a strong correlation between positive history of measles and seropositivity of measles antibody; however, Khare et al also note that roughly half of those with a negative history of measles are also seropositive. They speculate that this is likely due to subclinical infection as well as misdiagnosis but they also did not rule out the possibility of lingering maternal antibody effects in young children. Thus, seroprevalence studies are a very useful tool when developing a model of measles transmission since misdiagnosis and subclinical infection are not problematic. However, seroprevalence studies are generally not longitudinal, and only give a snapshot of seroprevalence at a given point in time.

A problem in many of the case report papers for both Africa and India was that cases were only reported in very young children in the older papers. In more recent papers, a fine age breakdown was not provided: this may reflect sample size issues, as measles tends to be more rare in more recent times, and case numbers tend to be smaller in rural populations (as was the case in many Indian studies). This caused difficulty in trying to discern any patterns or trends. Attack rates in older children are needed to accurately parameterize the force of infection in older children and thus more accurately describe herd immunity effects. Thus there is a need for studies that sample a larger population and at larger and finer age ranges.

For African populations, patterns in seroprevalence were difficult to determine due to the lack of available seroprevalence studies done in Africa. A caveat that appeared several times in the seroprevalence studies for both Africa and India was that not enough children were sampled in one or more age groups, giving an inaccurate view of seroprevalence within an age group. Another problem encountered in some African papers was that the percent seropositive would be given in an age group that spanned more than one age and so it was necessary to assume that the percent seropositive applied to the midpoint of the age range. This limited the number of samples that could be used to calculate the average seroprevalence in an age group. Finally, there was variation among the tests for antibody as to what level was considered positive for protective levels of antibody. Some studies reported the number of individuals exhibiting protective levels of antibody whereas others reported the number of individuals exhibiting any antibody; this clearly influences how exposure to the infection is inferred from reported percent seropositive at a given threshold.

Though the calculations for *R*_0 _for both India and Africa yielded values for the overcrowding exponent *c *that are similar to values found in population centres in England and Wales [20], their *R*^2 ^values and P-values suggest that the observed relation is not statistically significant. The large scatter observed in the plots may reflect unknown vaccination status in many children, limited data, lack of seroprevalence studies in Africa, and greater heterogeneity in populations included in the regression as compared to the relative homogeneity of populations across England and Wales.

## Conclusions

Here we have confirmed previous findings of significant differences in measles epidemiology in low-income versus developed countries, with a distinctly lower mean age at infection in African and Indian populations, where the age of peak attack rates occurs at age 1 or 2 years [14]. Thus, with the data available in the published literature, it would be possible to parameterize an informative dynamic model for measles in African and Indian populations. However, the quality of data in low-income countries still lags behind that in developed countries, and there is a need for more accurate epidemiological data in low-income countries. We were not able to show a statistically significant relationship between population density and the basic reproductive ratio *R*_0_, however, this may reflect other heterogeneities due to the broad sampling of populations used in the analysis. Finally, estimation of force of infection functions from age-stratified seroprevalence data suggest that the force of infection may decline monotonically with age in those populations, meaning that the eradication equation *p*_c _= 1-1/*R*_0 _may overestimate the required proportion of immune individuals for measles to be eradicated in those populations.

## Competing interests

The authors declare that they have no competing interests.

## Authors' contributions

EKS wrote the manuscript, conducted the literature review, and conducted part of the data analysis. LPG contributed to the study design and revised the manuscript. CTB conceived of and designed the study, conducted part of the data analysis, and revised the manuscript. All authors have read and approved the final manuscript.

## Authors' informations

EKS was an undergraduate research assistant at the time of writing. The manuscript co-authors are developing a model of measles transmission and vaccination in low-income countries.

## Supplementary Material

Additional file 1 **Reviewed case report studies for various countries in Africa**. This file contains a table that summarizes all included case report studies for Africa. Click here for file

Additional file 2 **Reviewed case report studies for various rural and urban areas in India**. This file contains a table that summarizes all included case report studies for India. Click here for file

Additional file 3 **Reviewed seroprevalence studies for various African cities**. This file contains a table that summarizes all included seroprevalence studies for Africa. Click here for file

Additional file 4 **Reviewed seroprevalence papers for various rural areas in India**. This file contains a table that summarizes all included seroprevalence studies for India. Click here for file
