# Supplementary material for: A review of data needed to parameterize a dynamic model of measles in developing countries
Source: BMC Res Notes. 2010 Mar 16;3:75. doi: 10.1186/1756-0500-3-75 (PMC2848058; doi:10.1186/1756-0500-3-75)
Supplement: Additional file 1 — Reviewed case report studies for various countries in Africa. This file contains a table that summarizes all included case report studies for Africa. [file 1756-0500-3-75-S1.DOC]

**Reviewed case report studies for various countries in Africa**

| **Study, Region, &**  **Population Size** | **Type,**  **Sample Size &**  **Year(s) of Study** | **Purpose/ Objective** | **Quantitative Results** | **Qualitative Results** | **Caveats** |
| --- | --- | --- | --- | --- | --- |
| Guyer and  McBean  1981 [46]^+^  Yaoundé, Cameroon  166,000 (1968)  260,000 (1975) | Case Reports -national surveillance -endemic  NA*  1968-1975 | Describe the epidemiology of measles in Yaoundé during the period prior to EPI (1968-1975) | Yearly attack rates per 1000 children 6-35mo:   | Year | Attack Rate | | --- | --- | | 1969 | 11 | | 1970 | 15 | | 1971 | 87 | | 1972 | 316 | | 1973 | 194 | | 1974 | 101 | | 1975 | 97 |   1971 attack rates per 1000 children in age group:   | Age (mo) | Attack rate | | --- | --- | | 0-5 | 8.9 | | 6-11 | 200.1 | | 12-23 | 95.6 | | 24-35 | 26.6 |   1975 attack rates per 1000 children in age group:   | Age (mo) | Attack rate | | --- | --- | | 0-5 | 4.7 | | 6-11 | 135.0 | | 12-23 | 73.0 | | 24-35 | 27.0 | | -SEP (1968) interrupted epidemic pattern seen previously but transmission still occurred at a much lower rate;  -surveillance improved in 1973-1975 but still a decrease in incidence;  -% cases has shifted slightly but significantly to older children in 1975 (herd immunity effect);  -theoretically enough vaccinations were given to children 6-35mo to provide adequate coverage of children in and entering susceptible population | -1968: cases seen at PMI Centrale were reported so only those cases treated at PMI were reported;  -1974: surveillance included all dispensaries and child health care centres, improved number reported cases  🡪 accuracy before this will be less accurate  🡪 still only reports treated cases  -incomplete records for 1969, 1970, last 6 months of 1972 |
| Heymann,  Maybean,  Murphy  et al 1983 [47]  Yaoundé, Cameroon  255,116 (1974)  278,076 (1975)  313,706 (1976)  342,939 (1977)  372,714 (1978)  406,258 (1979) | Case Reports -community surveillance  -endemic  40,349 (1975)  45,519 (1976)  49,615 (1977)  54,081 (1978)  58,948 (1979) -sample survey -endemic  187 (1976) 206 (1977) 209 (1979) | Describe the impact of the changes in the immunization policy in Yaoundé (1975-1979). This paper continues on from where Guyer et al (1981) leave off. | Incidence per 1000 population**:   | Year | Incidence | | --- | --- | | 1974 | 10.45 | | 1975 | 9.49 | | 1976 | 6.44 | | 1977 | 5.97 | | 1978 | 7.59 | | 1979 | 5.87 |   Incidence per 1000 children in age group (age in months):   |  | Age (mo) | | |  | | --- | --- | --- | --- | --- | | Year | 0-8 | 9-23 | 24-47 | Total | | 1975 | 88.2 | 92.7 | 34.6 | 65.4 | | 1976 | 36.9 | 78.8 | 23.3 | 44.4 | | 1977 | 31.1 | 71.3 | 24.7 | 41.2 | | 1978 | 37.6 | 82.5 | 37.9 | 52.3 | | 1979 | 31.8 | 64.5 | 27.5 | 40.5 | | -number of weekly reporting sites increased from 6 to 13 in 1977 but overall incidence still decreased over 1974-1979, except for a peak in 1978 -from 1975-1979 incidence decreased by 64% (0-8mo), 30.4% (9-23mo), 20.5% (24-47mo) -constant vaccination coverage of 39-40% (12-23mo) from 1976-1979 | -sample survey could have contained children from other age groups to determine possible second dose coverage -surveillance system will miss cases not treated at health facilities |
| Taylor,  Ruti-Kalisa,  Ma-Disu  et al 1987 [48]  Kinshasa, Zaire  3,000,000 | Case Reports -community survey  (3 months prior to survey)  3092  1983 | Describe epidemiology of measles to measure the effects of the vaccination program | Community survey: Incidence rate per 1000 children in age group (over 3 months)†:   | Age (mo) | Incidence | | --- | --- | | 0-5 | 0 | | 6-8 | 61.9 | | 9-11 | 67.0 | | 12-23 | 45.2 | | 24-35 | 11.8 | | 36-47 | 10.3 | | 48-59 | 3.7 | | Total | 21.7 |   -18% cases <9mo  -37% cases <1yr  Reported Cases:  -27% cases <9mo  -45% cases <1yr | -proportion of children 9-11mo vaccinated increased 1977-1984 even though same number vaccines were given -vaccine coverage increased from 37±6% (1977) to 62±4% (1983) in children 12-23mo -62% children in community survey vaccinated  -vaccination varied from 16-73% in different clusters 🡪non-uniform vaccine coverage within Kinshasa leaves pockets of susceptibles -27% cases <9mo 🡪 might be a reservoir for measles transmission because they are too young to be vaccinated but have the highest incidence rate -45% cases <1yr  -herd immunity was not evident with 50-60% coverage in Kinshasa | -community survey only done in 3 months prior to the survey  -children only assumed vaccinated if they have a vaccination card |
| Dollimore,  Cutts,  Binka  et al 1997 [49]  Kassena-Nankana, Ghana  180,000 | Case Reports -field survey -endemic  25,443  1989-1991 | Report on a community-based study in northern Ghana that studied CFRs, incidence, and effects of vitamin A supplementation | Incidence per 1000 child-years of follow up:   | Age (mo) | Incidence | | --- | --- | | 0-5 | 9.9 | | 6-8 | 46.2 | | 9-11 | 31.4 | | 12-23 | 22.1 | | 24-35 | 23.7 | | 36-47 | 23.3 | | 48-59 | 24.6 | | 60-71 | 25.3 | | ≥72 | 23.1 | | Total | 24.3 |   -incidence was 35.5 per 1000 child-years among unvaccinated children and 10.7 per 1000 child-years among vaccinated children | -median age at onset: 41mo -lower vaccination 🡪 higher incidence -no vitamin A supplements 🡪 higher incidence (not stat. sig) -vaccine coverage was ~50% for entire study period  -median age of vaccination was 11.7 months | -visits were only every 4 months 🡪possibility for inaccurate reporting -exact date of measles onset not recorded 🡪possibility that cases were recorded in wrong age group but it was over 2yrs |
| Weeks, Barenzi,  and Wayira 1992 [42]  Kampala, Uganda  800,00 | Case Reports -household survey -outbreak  301  1990 | Assess the issue of the large proportion  of cases that occurred in children that have been vaccinated | All cases were within 5mo to 12 yrs:  -4% cases <9mo  -75% cases <5yr  -25% cases >4yr Attack rates per 1000 children in age group‡:   | Age (mo) | Attack rate | | --- | --- | | <9 | 55.6 | | 9-11 | 50.0 | | 12-23 | 320.5 | | 24-35 | 140.4 | | 36-47 | 150.0 | | 48-59 | 153.8 | | Total | 169.4 |   -40% children aged 12-23mo in zone 1 and 58% in zone 2 were vaccinated   | Age (mo) | % vaccinated | | --- | --- | | 9-11 | 45 | | 12-23 | 47 | | 24-35 | 58 | | 36-47 | 38 | | 48-59 | 48 | | Total | 48 | | -vaccine efficacy varies depending on zone:  75% in zone 1, 55% in zone 2  -no major problems with the cold chain were found but improved maintenance and stock control need to be improved | -zones were chosen that had the highest number of reported cases -case detection may have been influenced by mother’s recall or by incorrect diagnosis -two communities did not describe the extent of the outbreak or immunization coverage  -small sample size considering the size of the city |
| Malfait, Jataou,  Jollet et al 1994 [43]  Niamey, Niger  445,715 (1988) | Case Reports -national surveillance -outbreak  435,606  1990-1991 | Describe the epidemiological characteristics, risk factors for illness, recommendations developed during the outbreak, and calculate vaccine efficacy | Incidence rates per 1000 children in age group for epidemic (7 months)§:   | Age (mo) | Incidence | | --- | --- | | 0-5 | 46.4 | | 6-8 | 279.1 | | 9-11 | 244.5 | | 12-59 | 105.1 | | 5-14 | 18.2 | | ≥15 | 4.8 | | Total | 31.2 | | -vaccine coverage in Niamey was estimated to be 73% in children 12-23mo and 63% in children 9-11mo in 1990  -highest IR in age group 6-8mo then 9-11mo 🡪 need vaccine with higher efficacy at 6mo, however, sufficient vaccine coverage levels in older children would have reduced risk in younger children (herd immunity) -low vaccine efficacy seems not to be the cause of the epidemic | -bias in control group for calculating VE since they could be more likely to attend health facilities often  -controls also had no history of measles infection |
| Grais, Dubray,  Gerstl et al 2007 [44]  Boukoki, Niamey, Niger,  NA  Moursal, N'Djamena, Chad,  NA  Dong District, Adamawa State, Nigeria  NA | Case Reports -household  -retrospective survey -outbreak  26,795  (Niger) 21,812  (Chad) 16,249  (Nigeria)  2003-2005 | Estimate measles ARs, CFRs, frequency of measles (and related complications), and access to health care during these epidemics in order to provide a picture of measles morbidity and mortality in high burden settings | Boukoki attack rates per 1000 children in age group:   | Age (yrs) | Attack rate¶ | | --- | --- | | <5 | 170.6 | | 5-14 | 23.3 | | ≥15 | 2.3 | | Total | 38.2 |   Moursal:   | Age (yrs) | Attack rate¶ | | --- | --- | | <5 | 171.7 | | 5-14 | 37.1 | | ≥15 | 2.4 | | Total | 34.2 |   Dong District:   | Age (yrs) | Attack rate¶ | | --- | --- | | <5 | 243.3 | | 5-14 | 115.3 | | ≥15 | 10.6 | | Total | 87.9 |   Vaccine coverage:  Boukoki: 37.3%  Moural: 70%  Dong District: 1.0% | -Dong District in Nigeria had highest AR of all 3 districts -Nigeria also had the lowest vaccine coverage -few cases below 9mo in all 3 districts -18mo median age of case fatality -visitiation of health centre lowest in Nigeria  -money was a factor in all 3 places  -treatment was not free  -geographical limitations | -poor age breakdown for ARs -study during epidemic -survey was conducted 6mo after epidemic started so cases at beginning may not have been reported -site selection biased towards more accessible areas -ages are imprecise |

+ Reference numbers in brackets refer to reference list in main manuscript text.

*NA = Not Available

**values were changed from incidence per 100000 to incidence per 1000

†values recalculated to be incidence per 1000 rather than per 100

‡ values recalculated to be incidence per 1000 rather than per 100

§ original IR values were per 1000 person months; number of people per age group was found by dividing by length of epidemic (7 months)

¶ values recalculated to be incidence per 1000 rather than per 100
