# Supplementary material for: A review of data needed to parameterize a dynamic model of measles in developing countries
Source: BMC Res Notes. 2010 Mar 16;3:75. doi: 10.1186/1756-0500-3-75 (PMC2848058; doi:10.1186/1756-0500-3-75)
Supplement: Additional file 2 — Reviewed case report studies for various rural and urban areas in India. This file contains a table that summarizes all included case report studies for India. [file 1756-0500-3-75-S2.DOC]

**Reviewed case report studies for various rural and urban areas in India**

| **Study, Region, &**  **Population Size** | **Type,**  **Sample Size &**  **Year(s) of Study** | **Purpose/ Objective** | **Quantitative Results** | **Qualitative Results** | **Caveats** |
| --- | --- | --- | --- | --- | --- |
| Garai and  Chakraborty  1980 [50]^+^  40 villages in West Bengal  57,267 | Case Study -house-to-house study -endemic  57,267  1976-1978 | Study epidemiological features of measles, describing incidence in relation to age, sex and season in rural community | Annual incidence per 1000 children in age group:   | Age (yrs) | Incidence | | --- | --- | | <6mo | 9.1 | | 6-11mo | 55.3 | | 1 | 37.9 | | 2 | 20.8 | | 3 | 14.4 | | 4 | 12.8 | | 5-9 | 9.8 | | 10-14 | 3.8 | | 15-19 | 1.4 | | 20-29 | 0.9 | | 30-39 | 1.3 | | 40+ | 0.1 | | Total | 5.0 |   -12.2% cases below 1 year  -50% cases by age 5  -90% cases by age 14 | -1977 seems to be an epidemic year  -epidemics occur every 2-3 years | -incidence rates per year could have been useful in determining epidemic year |
| Bhaskaram,  Radhakrishna,  and Madhusudan  1986 [52]  slum of Hyderabad city  NA | Case Study -endemic  1544  1982-1983 | Determine minimum age for routine vaccination,  maximum age for vaccination to control the disease, and optimum age for vaccination (ie. when vaccination would be most beneficial) when there is no immediate threat of infection | Min. Age: -all cord and maternal antibodies sufficiently high -all infants ≤3mo have protective antibodies -60% 4-9mo had protective antibodies, 8% of which were 9mo Max. Age: -1006/1544 clinically susceptible --90% of infants --63.3% 1-3yrs --51.2% of 3-5yrs   | Age (yrs) | Incidence (%) | | --- | --- | | 0-1 | 11.7 | | 1-2 | 17.7 | | 2-3 | 11.7 | | 3-4 | 8.0 | | 4-5 | 9.0 |   -50% cases occurred below 2yrs --21.3% of these were infants -95% susceptibles get measles before 5yrs Reduce to 6mo?: -no children <3mo had measles -34.8% infants affected 4-9mo -65.2% '' '' 10-12mo Vaccine Failure: -17.1% in 9-12mo -42.1% in 13-15mo -->unclear why this is -2% in 15-36mo | Min Age:  9mo min age for routine vaccination  Max Age:  -90-95% coverage needed before 5 yrs  Reduce to 6mo?:  -no need to vaccinate earlier than 9mo due to herd immunity  Vaccine Failure:  -optimal age to vaccinate would be 15 mo for children not at immediate risk | -study period was only over one year which may or may not have been an epidemic year |
| Jain, Meena,  Yadav et al 1990 [51]  Ramgarh village  5923 | Case Study  -endemic  2200  1986-1988 | Describe the epidemiology of measles in rural India | Attack rate per year per 1000 children in age group:   | Age (yrs) | Attack rate | | --- | --- | | 0-1 | 26.5 | | 2-3 | 51.1 | | 4-5 | 58.8 | | 6-7 | 27.9 | | 8-9 | 10.6 | | ≥10 | 4.6 | | Total | 31.5 |   -46.2% of children with no history of measles infection or vaccination were seropositive | -seropositivity in children with no history of measles infection or vaccination higher in this study than in Sehgal et al (1983) and Bhardwaj et al (1988) | -no mention of vaccination history in children surveyed for measles infection |
| Pereira and Benjamin 1972 [53]  Sathuvachari village  1200 | Case Study -outbreak  194  1969 | Observe mortality and morbidity of an epidemic in a small village | Attack rates per 1000 children in age group:   | Age (yrs) | Attack rate* | | --- | --- | | 0-1 | 250.0 | | -2 | 555.6 | | -3 | 840.0 | | -4 | 687.5 | | -5 | 250.0 | | -6 | 121.1 | | Total | 433.0 | | -132/194 of the children attended a feeding centre  -41 children attending centre had previous immunization; 2 developed measles -33 had no immunization; 23 developed measles -31 ages 5-7yrs not attending centre previously immunized; no measles | -small study population  -some children had better nutrition than others due to feeding centre |
| Jajoo, Chhabra,  Gupta et al 1984 [54]  Barbadi village  1434 | Case Study -outbreak  192  1982 | Provide information on measles incidence and rate of complications for a rural area of India since not many of these studies have been done | Attack rate per 1000 in age group:   | Age (mo) | AR in susceptiblepop† | AR in total pop‡ | | --- | --- | --- | | 0-5 | 125.0 | 125.0 | | 6-8 | 714.3 | 714.3 | | 9-11 | 900.0 | 818.2 | | 12-23 | 545.5 | 450.0 | | 24-35 | 807.7 | 567.6 | | 36-47 | 764.7 | 361.1 | | 48-59 | 700.0 | 241.4 | | Total | 570.4 | 401.0 | | -youngest case was 4mo -oldest case was 10yrs -135/192 underfives were susceptible at the beginning of the outbreak; 77 caught measles during epidemic  -few cases consulted treatment  -by age of 10yrs, almost all children had measles; 50.5% before 4^th^ birthday | -small population per age group though this is not the fault of the authors |
| Sharma, Kaushic, Johri et al 1984 [55]  6 villages investigated, only 2 with measles: -Karanda -Bahaduri  2,769 | Case Study -outbreak  238  1982 | Investigate a measles epidemic involving 2 villages in Alwar district | Attack rate per age group:   | Age (yrs) | Attack Rate (%) | | --- | --- | | <1 | 35.1 | | 1 | 72.2 | | 2 | 49.0 | | 3 | 33.3 | | 4 | 34.2 | | >5 | 36.4 | | Total | 42.8 | |  | -small population per age group though this is not the fault of the authors |
| Narain, Khare, Rana et al 1989 [56]  13 villages Pilkhi Primary Health Centre  56,000 | Case Study -outbreak  4542  1986 | Describe the epidemiology of a large outbreak in Tehri Garhwal district, Uttar Pradesh | Attack rates per age group. Also given is the range of ARs over all 13 villages:   | Age (yrs) | AR (%) | Range | | --- | --- | --- | | <5mo | 13.8 | NA | | 6-8mo | 21.1 | NA | | 9-11mo | 50 | NA | | <1 | 22.4 | 0-100 | | 1-4 | 54.5 | 3.3-100 | | 5-9 | 46.2 | 10.3-100 | | 0-9 | 45.3 | 5.2-96.9 | | 10-14 | 45.3 | 0-95.2 | | 15+ | 1.1 | 0-15.4 | | Total | 16.9 | 1.5-54.3 |   -38% cases below 5yrs -76% below 10yrs | -spread of measles was very rapid  -last outbreak in 1982 in 4 villages  -no vaccination  -youngest case 5mo | -poor age breakdown -some villages may not be suitable to use due to very small population and no contact with other villages |
| Risbud, Prasad, Mehendale  et al 1993 [57]  2 hamlets of Vavar village,  Mokhada Taluk,  Thane district,  Maharashtra: -Behedpada  593  -Sagpanipada  270 | Case Study -house to house survey -outbreak  94 (Sagpanipada) 268 (Behedpada)  1992 | Report on the epidemic which occurred while world wide efforts to eradicate measles were taking place | Attack rates (%) for each hamlet and overall:   | Age (mo) | Ham #1 | Ham#2 | Total | | --- | --- | --- | --- | | 0-9 | 30.0 | 18.7 | 23.1 | | 10-12 | 50.0 | 75.0 | 67.6 | | 13-24 | 52.3 | 81.1 | 70.7 | | 25-36 | 61.9 | 77.1 | 71.4 | | 37-48 | 72.7 | 74.2 | 73.8 | | 49-60 | 36.4 | 36.4 | 36.4 | | 61-72 | 66.7 | 52.6 | 54.5 | | 73-120 | 50.0 | 9.3 | 12.1 | | Total | 52.7 | 51.4 | 51.8 |   -all cases <10yrs -~96% cases <6yrs | -no record of vaccination was available for these hamlets although one vaccinator used to visit the hamlets to vaccinate against polio, triple, and measles  -CFR seen in Sagpanipada highest from any study reported so far | -small population per age group though this is not the fault of the authors |
| Gupta and Sharma 2006 [58]  remote village of Himachal Pradesh/  NA | Case Study -house to house survey -outbreak  1360 (2004) | Provide info on a more recent outbreak in India | Attack rates:   | Age (yrs) | AR (%) | | --- | --- | | 0-4 | 1.7 | | 5-9 | 19.4 | | 10-15 | 34.5 | | Total | 16.2 | | -no infant or adult affected with measles -no deaths due to measles reported -71.3% children <15yrs were unvaccinated -of those vaccinated, all were under 9yrs -no case of measles occurred in those vaccinated | -vaccine record dependent of parental recall -poor age breakdown -small population per age group though this is not the fault of the authors |

+ Reference numbers in brackets refer to reference list in main manuscript text.

*calculated from data given in paper: number with measles/total number per age group

†original values were given as percents %

‡calculated from data given in paper: number of measles cases/population per age group
