# Supplementary material for: A review of data needed to parameterize a dynamic model of measles in developing countries
Source: BMC Res Notes. 2010 Mar 16;3:75. doi: 10.1186/1756-0500-3-75 (PMC2848058; doi:10.1186/1756-0500-3-75)
Supplement: Additional file 3 — Reviewed seroprevalence studies for various African cities. This file contains a table that summarizes all included seroprevalence studies for Africa. [file 1756-0500-3-75-S3.DOC]

Reviewed seroprevalence studies for various African cities

| **Study, Region &**  **Population Size** | **Type,**  **Sample Size &**  **Year(s) of Study** | **Purpose/ Objective** | **Quantitative Results** | **Qualitative Results** | **Caveats** |
| --- | --- | --- | --- | --- | --- |
| Stanfield and  Bracken 1971 [63]^+^  Kampala area, Uganda  NA | SE* study  352  NA** | Investigate the means by which measles vaccine could be used economically and reliably in developing countries | | Age (mo) | # +ve/  total | % +ve | | --- | --- | --- | | 1-3 | 108/130 | 83.1 | | 4-5 | 3/11 | 27.3 | | 7-11 | 8/42 | 19.0 | | 12-35 | 55/117 | 47.0 | | 36-71 | 31/39 | 79.4 | | ≥72 | 9/13 | 69.2 | | Total | 214/352 | 60.8 |   -246 cord blood samples: 85.4% seropositive (210/246) |  | -focus of paper was not on seroprevalence so no discussion of this is given |
| Munube 1979 [38]  Busoga District, Uganda  NA | SE study  319  1972 | To better understand and evaluate the importance of the situation where people believe it's possible to acquire measles more than once | | Age | +ve/  total | % +ve | | --- | --- | --- | | 0-11mo | 2/9 | 22.2 | | 1yr | 12/33 | 36.4 | | 2yr | 45/66 | 68.2 | | 3yr | 55/74 | 74.3 | | 4yr | 61/70 | 87.1 | | 5yr | 51/60 | 85.0 | | 6yr | 7/7 | 100 | | -76% of children with positive measles history were actually seropositive -50% of children with negative measles history were actually seropositive -->other diseases with similar symptoms cause confusion and misdiagnosis -->many people believe it's possible to acquire measles more than once -->this may cause parents to not take their children to get vaccinated if they see no point | -no mention of vaccine history or medical treatment -because people believe that a person can acquire measles more than once, they don't see the point of vaccination -this shows that case report studies may not be that reliable due to inaccurate reporting (overreporting due to recurrence beliefs and misdiagnosis) |
| Ogunmekan,  Bracken, and  Marshall 1981 [64]  Lagos, Nigeria  NA | SE study  592  NA | Determine prevalence of measles among a group of children, some blind and/or deaf, in order to assess the significance of measles in causing these handicaps | Cord blood seroprevalence: 100%   | Age | % +ve | | --- | --- | | <5mo | 42 | | 5-6mo | 36 | | 7-8mo | 5 | | 9-10mo | 15 | | 11-12mo | 22 | | 1-5yrs | 53 | | 6-10yrs | 75 | | 11yrs | 100 | | 16yrs | 100 | | 21+yrs | 100 | |  | -infants tested were those with access to a health clinic -sample size of children is small -majority of them are in the age group 9-10mo |
| Tolfvenstam  Enbom,  Ghebrekidan et al 2000 [62]  Massawa, Eritrea  NA | SE study  411  1995 | Investigate seroprevalence of measles in Eritrea populations to determine high risk groups | | Age | +ve/  total | % +ve | | --- | --- | --- | | <1yr | 25/29 | 86 | | 1-5yrs | 41/48 | 85 | | >5yrs | 78/84 | 93 | | -immunization program: ~29% coverage by 1995 🡪no data on immunization for this particular study group | -small sample size -children above 5 sampled were those attending health stations to receive food aid -poor age breakdown -discussion is minimal -vaccine coverage is not mentioned |
| Enquselassie  Ayele,  Dejene et al 2003 [37]  Addis Ababa, Ethiopia  2,100,000 | SE study  4654  1994 | Estimate FOI acting on susceptibles in a population with a vaccination programme in place | -FOI estimate: 23% -prop. susceptible: <5 - 24% <15 - 9%   | **Age** | **Pop** | **% +ve*** | | --- | --- | --- | | 0-11mo | 55 | 57 | | 1 | 69 | 75 | | 2 | 69 | 78.5 | | 3 | 100 | 73 | | 4 | 124 | 85 | | 5 | 108 | 86.5 | | 6 | 90 | 87.5 | | 7 | 141 | 95 | | 8 | 125 | 93 | | 9 | 145 | 97 | | 10 | 175 | 97 | | 11 | 114 | 96 | | 12 | 178 | 97 | | 13 | 155 | 95 | | 14 | 157 | 97.5 | | 15-19 | 890 | 97.5 | | 20-29 | 985 | 99.5 | | 30-39 | 599 | 99.9 | | 40-49 | 375 | 99.9 | |  | **4654** |  | | -important assumptions:  🡪effect on seroprevalence by measles vaccination has been constant from 1980-84 🡪any changes in proportion seronegative are assumed to be the result of measles transmission very few people above 14yrs will have been vaccinated -few false positive reports of measles but high proportion of individuals failed to recognize or remember they had measles 🡪 historical info is unreliable in identifying at-risk individuals -fig 4 assumes constant FOI 🡪allowing for diff. FOI values for diff. age groups gives no sig. improvement in the fig. |  |

+ Reference numbers in brackets refer to reference list in main manuscript text.

*SE = Seroepidemiological

**NA = Not Available

**†**estimated from Fig. 1 in paper
