# Supplementary material for: A review of data needed to parameterize a dynamic model of measles in developing countries
Source: BMC Res Notes. 2010 Mar 16;3:75. doi: 10.1186/1756-0500-3-75 (PMC2848058; doi:10.1186/1756-0500-3-75)
Supplement: Additional file 4 — Reviewed seroprevalence papers for various rural areas in India. This file contains a table that summarizes all included seroprevalence studies for India. [file 1756-0500-3-75-S4.DOC]

**Reviewed seroprevalence papers for various rural areas in India**

| **Study, Region &**  **Population Size** | **Type,**  **Sample Size &**  **Year(s) of Study** | **Purpose/ Objective** | **Quantitative Results** | **Qualitative Results** | **Caveats** |
| --- | --- | --- | --- | --- | --- |
| Mehta, Nanavati, Jhala et al 1972 [65]^+^  Bombay  NA* | SE** study  650  NA | Obtain information regarding transplacental transfer of measles antibodies, persistence of these antibodies during infancy, and age of onset of measles | Seroprevalence by age:   | Age | % positive | | --- | --- | | 1 mo | 93.3 | | 2 “ | 87.5 | | 3 “ | 75 | | 4 “ | 55.5 | | 5 “ | 33.3 | | 6 “ | 25 | | 7 “ | 0 | | 8 “ | 0 | | 9 “ | 12.5 | | 10 “ | 14.3 | | 11 “ | 16 | | 1 yrs | 25 | | 2 “ | 33.3 | | 3 “ | 33.3 | | 4 “ | 48 | | 5 “ | 72 | | 6 “ | 85.7 | | 7 “ | 100 | | 8 “ | 100 | | 9 “ | 70 | | 10 “ | 80 | | 11 “ | 81.8 | | Adults | 78 | | -definite correlation btw presence of antibodies and history of measles | -some age groups have small population |
| John and Jesudoss 1973 [66]  Vellore Town  NA | SE study  277  NA | Present evidence of high prevalence of measles among the very young children | | Age (yrs) | % positive† | | --- | --- | | 0-3mo | 83.3 | | 3-6mo | 25 | | 6-9mo | 33.3 | | 9-12mo | 66.7 | | 1 | 51.1 | | 2 | 67.3 | | 3 | 76.5 | | 4 | 82.9 | | 5 | 87.5 | | 6 | 76.5 | | 7 | 77.8 | | 8 | 75 | | 9 | 100 | | -median age of measles was below 24mo | -small pop in upper age groups and infants |
| Broor, Pal, Banerjee et al 1976 [67]  Chandigarh area  NA | SE study  568  NA | Determine seroprevalence and incidence of past measles infection in different age groups | | Age (yrs) | % positive | | --- | --- | | 0-4 | 46.7 | | 5-9 | 77.3 | | 10-14 | 87.8 | | 15-24 | 95.9 | | 25-34 | 99.0 | | 35+ | 100 |   Overall %: 84.7 | -preschool and young school going ages have rapid acquisition of measles  -incidence of measles in India is comparable to that in Western countries | - 0-4 yr age group further broken down but confusing |
| Bhau, Madhavan and Agarwal 1979 [68]  Pondicherry  NA | SE study  350  NA | Assess the immune status of children in the Pondicherry area since not many serological studies have taken place | | Age (yrs) | % positive | | --- | --- | | 0-6mo | 55 | | 6-12mo | 26.08 | | 1 | 57.15 | | 2 | 50 | | 3 | 51 | | 4 | 75.8 | | 5 | 78.5 | | 6 | 89.1 | | 7 | 87.9 | | 8-10 | 76 | | 10-12 | 85.3 | | -preschool and young school going ages have rapid acquisition of measles |  |
| Sehgal, Sharma, Mehta et al 1983 [69]  Delhi and Alwar  NA | SE study  939  NA | Obtain information on the past incidence of measles virus infection in different age groups of the population | | Age (yrs) | % positive | | --- | --- | | <6mo | 0 | | 6-11mo | 8.34 | | 1 | 17.31 | | 1.5 | 36.85 | | 2 | 34.93 | | 2.5 | 41.67 | | 3 | 42.69 | | 3.5 | 45.46 | | 4 | 45.08 | | 4.5 | 54.55 | | 5 | 62.50 | | 5.5 | 100 |   -3.1% of maternal blood seronegative -100% cord blood seropositive |  | -never specified whether the cord blood came from the same mothers; if so, how can the cord blood be 100% but mothers only be 96.9%? -sero above 5 years useless/nonexistent; no point including it in the study |
| Khare, Dutta, Kumari  et al 1987 [70]  Delhi  NA | SE study  369  1984-1985 | Evaluate reliability of past history of measles infection and determine frequency of subclinical infection | | Age (yrs) | % positive | | --- | --- | | 1 | 100 | | 2 | 66.6 | | 3 | 60 | | 4 | 50 | | 5 | 50 | | 6 | 64.7 | | 7 | 10 | | 8 | 35.71 | | 9 | 35.3 | | 10 | 35.7 | | 11 | 38.5 | | 12 | 41.86 | | 1 | 47.27 | | 2 | 74.28 | | 3 | 78.26 | | 4 | 73.68 | | 5 | 83.33 | | >6 | 84.78 |   -0/182 <1yr had history of measles -60/187 >1yr had history of measles | -all 182 children below 1 yr gave no history of measles  -60/187 above 1 yr had previous measles infection  --close correlation between measles history and seropositivity (55/60)  -of those with no measles history, 57 (44.8%) were seropositive |  |

+ Reference numbers in brackets refer to reference list in main manuscript text.

*NA = Not Available

**SE = Seroepidemiological

†values recalculated to include more decimal places
